# Proteomic Analysis of the Ontogenetic Variability in Plasma Composition of Juvenile and Adult *Bothrops jararaca* Snakes

**DOI:** 10.1155/2013/135709

**Published:** 2013-04-22

**Authors:** Karen de Morais-Zani, Kathleen Fernandes Grego, Aparecida Sadae Tanaka, Anita Mitico Tanaka-Azevedo

**Affiliations:** ^1^Laboratório de Herpetologia, Instituto Butantan, Avenida Vital Brazil 1500, 05503-900 São Paulo, SP, Brazil; ^2^Programa de Pós-Graduação Interunidades em Biotecnologia, Universidade de São Paulo, Avenida Professor Lineu Prestes 2415, 05508-900 São Paulo, SP, Brazil; ^3^Departamento de Bioquímica, Universidade Federal de São Paulo, Rua Três de Maio 100, 04044-020 São Paulo, SP, Brazil

## Abstract

The ontogenetic variability in venom composition of some snake genera, including *Bothrops*, as well as the biological implications of such variability and the search of new molecules that can neutralize the toxic components of these venoms have been the subject of many studies. Thus, considering the resistance of *Bothrops jararaca* to the toxic action of its own venom and the ontogenetic variability in venom composition described in this species, a comparative study of the plasma composition of juvenile and adult *B. jararaca* snakes was performed through a proteomic approach based on 2D electrophoresis and mass spectrometry, which allowed the identification of proteins that might be present at different levels during ontogenetic development. Among the proteins identified by mass spectrometry, antihemorrhagic factor Bj46a was found only in adult plasma. Moreover, two spots identified as phospholipase A_2_ inhibitors were significantly increased in juvenile plasma, which can be related to the higher catalytic PLA_2_ activity shown by juvenile venom in comparison to that of adult snakes. This work shows the ontogenetic variability of *B. jararaca* plasma, and that these changes can be related to the ontogenetic variability described in its venom.

## 1. Introduction

Poisonous snakes are responsible for around 50,000 deaths among five million cases of ophidian accidents per year in the world, especially in the rural areas of tropical countries in Asia, Africa, and South America [1, 2].

Envenomation by Viperidae snakes causes local tissue damages such as edema, hemorrhage, and myonecrosis, which are not well neutralized by conventional antivenom serotherapy [3]. *Bothrops jararaca *(*B. jararaca*) snake belongs to the Viperidae family and is the main reason for ophidian accidents in the state of São Paulo, Brazil [4]. Its victims usually have, besides systemic reactions of envenomation such as bleeding and blood incoagulability, local effects at the bite site such as edema, ecchymoses, compartmental syndrome, blisters, and necrosis [5]. The envenomation symptomatology has always stimulated researches on snake venom composition and function.

Unfortunately, the same is not observed for snake plasma. Despite extensive biochemical and molecular characterization of blood coagulation in mammals, little information is available about haemostasis in other vertebrates [6], although there is an increasing interest in the “natural resistance” of snakes towards the toxicity of its own venom and towards other snake venoms. The inter- and intraspecies resistibility can contribute to the development of new strategies for the treatment of snake envenomation and the discovery of proteins that can neutralize the toxic components of these venoms [7], making snake plasma a rich source of bioactive molecules.

It has been proposed that there are two different mechanisms that may account for this “natural immunity” [8]: (i) mutation of the gene encoding the target of the venom toxin, providing target resistance to the toxin [9–12] or (ii) presence of proteins that neutralize hemorrhagins [13–16], neurotoxins [17–22], or myotoxins [23, 24] in the blood of resistant animals. Several studies have shown that these proteins are either metalloproteinase inhibitors (antihemorrhagic factors) or PLA_2_ inhibitors (PLIs) (antineurotoxic/antimyotoxic factors) [8, 25–28].

Our group has purified and characterized two proteins from the plasma of *B. jararaca *snake, probably involved in its self-defense against accidental envenomation: (i) BjI, a blood coagulation inhibitor that recognizes thrombin-like enzymes present in *B. jararaca *venom by western blotting, suggesting a protective role of this protein [29] and (ii) fibrinogen [30], which showed resistance to hydrolysis caused by snake venoms. Interestingly, while bovine thrombin coagulated both *B. jararaca *and human fibrinogen, *B. jararaca *venom clotted human fibrinogen, but not *B. jararaca *fibrinogen. In addition, *C. durissus terrificus *and *Lachesis *sp. venoms could also clot human fibrinogen, with no action upon *B. jararaca *fibrinogen [31].

Another interesting feature described in some snake species is the ontogenetic variability in venom composition, a well-documented phenomenon that has long been studied [32]. Ontogenetic variation in venom composition has been reported in a number of genera [33–37], including *Bothrops *snakes [32, 38–41], which accounts for the differences in the clinical manifestations and severity of envenomation by juvenile and adult* B. jararaca *[42].

All the peculiarities related to ontogenetic variation in *B. jararaca *venom raised the question of whether the plasma composition of snakes follows the same modifications described in the venom. Therefore, a comparative study of the plasma composition of juvenile and adult *B. jararaca *snake was carried out. The present study focused on the antivenom proteins, considering their importance for the self-protection of these animals and for the search of new proteins for antivenom treatment.

## 2. Material and Methods

### 2.1. Blood Collection

**Figure 1:**
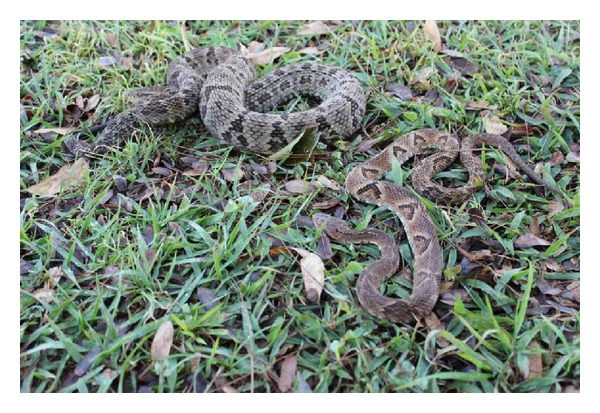
Adult and juvenile *B. jararaca *specimens.

Six specimens of *B. jararaca *(3 juveniles and 3 adults) from the Laboratory of Herpetology, Butantan Institute, São Paulo, Brazil, were used for this analysis. All specimens were females, juveniles (<60 cm snout-vent length) or adults (>82 cm snout-vent length) [43, 44] (Figure 1). Blood was collected by caudal venipuncture. Citrated blood samples were collected in a 9 : 1 ratio of blood to 3.8% sodium citrate solution, and plasma was obtained by blood centrifugation for 15 min at 1,200 g at room temperature and stored at −20°C. The Committee for the Ethical Use of Animals of Butantan Institute approved the experimental protocols (Protocol no. 542/08).

### 2.2. Protein Determination

Protein concentrations were determined using bicinchoninic acid (Sigma, St. Louis, MO, USA) and bovine serum albumin (BSA) (Sigma, St. Louis, MO, USA) as a standard, according to Smith et al. [45].

### 2.3. Two-Dimensional Electrophoresis (2D Electrophoresis)

Two-Dimensional electrophoresis was used to separate proteins in the first dimension by isoelectric focusing (IEF) and in the second dimension by molecular weight using SDS-PAGE electrophoresis. IEF was carried out using precast Immobiline DryStrip gels pH 3–10 gradient (24 cm—IPG strip) using an IPGphor unit (GE Healthcare, Uppsala, Sweden). On each gel, 1 mg of protein was loaded. The IPG strip and sample were covered with Dry Strip Cover Fluid (GE Healthcare, Uppsala, Sweden) and run at constant voltage of 100 V for 12 h and 500 V up to the accumulation of 500 Vh, followed by gradient voltage from 500 to 1,000 V up to the accumulation of 800 Vh, another gradient voltage from 1,000 to 10,000 Vh up to the accumulation of 16,500 Vh, and constant voltage of 10,000 V up to the accumulation of 22,200 Vh. IEF was followed by a SDS-PAGE using 10% resolving gels, according to Laemmli protocol [46], and DALTsix system (GE Healthcare, Uppsala, Sweden). The gels were run at constant amperage of 15 mA and constant voltage of 80 V for 1 h and then constant amperage of 60 mA and constant voltage of 500 V. Protein spots were visualized using Coomassie Blue R350 staining procedure according to GE Healthcare (Uppsala, Sweden) protocol. Each sample was analyzed in triplicate. Image acquisition of gels was performed using the ImageScanner III densitometer (GE Healthcare, Uppsala, Sweden) and the gels were analyzed using ImageMaster Platinum 7.0 software (GE Healthcare, Uppsala, Sweden). The spots were quantified using the % of spot volume criterion, which is automatically calculated by the ImageMaster software. The match analysis was performed in an automatic mode, and further manual editing was performed to correct mismatched and unmatched spots. A criterion of *P* < 0.05 was used to define the significant difference when analyzing the paired spots between the two groups (*n* = 3) according to ANOVA.

### 2.4. Protein Identification

For identification of spots with quantitative variation by mass spectrometry, gel spots were excised and in-gel trypsin digestion was performed according to Shevchenko et al. [47]. An aliquot (4.5 *μ*L) of the peptide mixture was separated by C_18_ (100 *μ*m × 100 mm) RP-nano UPLC (nanoAcquity UPLC, Waters, Milford, MA, USA) coupled with a Q-TOF Ultima mass spectrometry (Waters, Milford, MA, USA) with a nanoelectrospray source at flow rate of 600 nL/min. The gradient condition was 15–90% acetonitrile in 0.1% formic acid over 10 min. The instrument was operated in the “top three” mode, in which one MS spectrum is acquired followed by MS/MS of the top three most-intense peaks detected. The resulting spectra were acquired using MassLynx v. 4.1 software, and the raw data files were converted into a peak list format (mgf) without summing the scans using Mascot Distiller 2.2.1.0, 2008, Matrix Science (Matrix Science Ltd., London, UK) and searched against nonredundant protein database (NCBI) using Mascot, with carbamidomethylation as fixed modification and oxidation of methionine as variable modifications, one trypsin missed cleavage and tolerance of 20 ppm for both precursor and fragment ions.

## 3. Results and Discussion

It is known that many venomous snakes are resistant to their own venoms and that this natural resistance is due to the neutralizing factors present in their plasma. In the last years, studies on animals that resist the action of snake venoms have led to the discovery and characterization of proteins responsible for this resistance. The result of these studies was the structural and functional characterization of protein inhibitors of hemorrhagic metalloproteinases, as well as myotoxic and neurotoxic PLA_2_ [7]. 

Although specific endogenous inhibitors for snake venom have been widely described in the literature and have been the subject of review articles [25, 48, 49], the correlation between venom and plasma ontogenetic development has not been reported yet.

**Figure 2:**
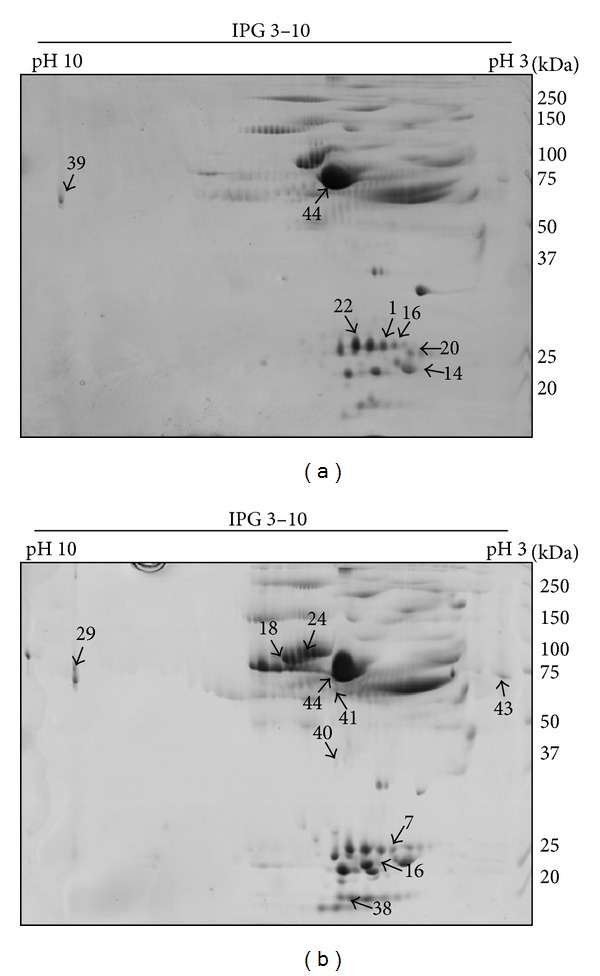
Analysis of juvenile and adult *B. jararaca *plasma by 2D electrophoresis. Plasma proteins (1 mg) from (a) juvenile and (b) adult were submitted to isoelectric focusing on 3–10 IPG strips (24 cm) followed by 10% SDS-PAGE. Gels were stained with Coomassie Blue R350. Spots present at different levels were indicated with arrows and identified by nanoESI-Q-TOF.

Plasma from juvenile and adult *B. jararaca* were analyzed by 2D electrophoresis and were compared using ImageMaster Platinum 7.0 software (see experimental section for details).  Figure 2 shows that the proteomic profile of juvenile and adult snakes is similar, suggesting minor ontogenetic differences between the plasma protein content of these two stages of development. The number of matches represents the spots identified in juvenile and adult plasma and exclusive spots were considered those present in only one group, juvenile or adult plasma. The results showed 1,250 matches between juvenile and adult plasma, with only 45 spots showing quantitative variation (*P* < 0.05). Taking into account these 45 spots, 18 are exclusive for juvenile and 16 for adult snakes. In addition, 5 spots were increased in juvenile and 6 in adults (Table 1), suggesting that the ontogenetic development is associated to little changes in the protein content of the plasma.

In order to identify spots present in different levels and correlate these differences to the snake development, the corresponding spots were excised, in-gel digested with trypsin, and submitted to mass spectrometry (MS/MS) (Table 2 and Figure 2). It is important to emphasize that only spots showing quantitative variation were submitted to MS/MS analysis. In addition, out of 45 spots analyzed, only 17 were identified, 7 in juvenile and 10 in adult plasma. During this process, we faced the limited available information about reptilian genome and proteome, described by other authors [50, 51]; thus, this study identified proteins by sequence homologies through the National Center for Biotechnology Information database (NCBI).

Among the proteins identified, transferrin was classified as increased in adult plasma (spot no. 18). This could be due to a differential iron transport mechanism across the development stage of snakes, as also reported for humans [52].

The complement system of snakes is of particular interest because the venom of *Naja naja *and related Asian snakes of the genus *Naja *[53] and the venom of *Austrelaps superbus *[54], an Australian elapid, contain a C3 structural and functional analog, cobra venom factor (CVF). Functionally, CVF resembles the C3 activation product C3b as it forms a complex with B factor in the presence of Mg^2+^ [55]. CFV and its analogs have become an important research tool in order to study the role of complement in host defense, immune response, and disease pathogenesis [53]. 

In *B. jararaca *plasma, C3 complement was identified in juvenile and adult plasma (Figure 2—spots 39 and 29, resp.) with a slight difference concerning the molecular weights. MS/MS analysis identified these spots as C3 complement by sequence homology to C3 from *Naja naja* (gi *|*399269). This protein, described in *Naja naja*, has molecular weight of 185 kDa and theoretical pI of 5.9. In this work, the two spots identified as C3 complement have molecular mass around 75 kDa and pI around 10, suggesting the presence of fragments in our samples.

Another protein present in adults, according to analysis by 2D electrophoresis, is the antihemorrhagic factor Bj46a (Figure 2—spot 41). Bj46a is a glycoprotein isolated from *B. jararaca *plasma that inhibits the snake venom metalloproteinases atrolysin C and jararhagin and *B. jararaca *venom hemorrhagic activity [56]. Interestingly, Antunes et al. [57] demonstrated that the venom of adult *B. jararaca *specimens was more hemorrhagic than the venom of newborn snakes. The hemorrhagic activity present in *B. jararaca* venom is generally credited to P-III metalloproteinases, like jararhagin [58, 59]. The reduced hemorrhagic activity present in the newborn *B. jararaca* venom described by Antunes et al. [57] appears to be correlated with the lack of jararhagin in newborn venom. This work showed the sequence of about 35% of Bj46a (data not shown). All of the 122 amino acids identified showed identity to the corresponding sequence present in the databank. However, Bj46a was also identified in juvenile *B. jararaca* plasma submitted to 1D electrophoresis and analyzed by Fourier Transform Ion Cyclotron Resonance mass spectrometry (data not published), suggesting the presence of this inhibitor in juvenile and adult snakes. One hypothesis to explain this finding is that Bj46a might be present in low levels in juvenile *B. jararaca* plasma and could not be identified by 2D electrophoresis. This finding linked to our results suggests a correlation between the ontogenetic development of the venom and the plasma composition of *B. jararaca*.

The high incidence of PLI identified among spots with quantitative variation is noteworthy. This protein corresponds to 71 and 30% of the total proteins identified in juvenile and adult plasma, respectively, and the present work showed that PLIs are increased in juvenile snakes (Figure 2—spots, nos. 16 and 22).

Forty-nine percent of *γ*-PLI sequence was obtained in this study (data not shown). Out of 99 amino acids identified, only one has no identity to the corresponding sequence present in the databank. This is the first time that this protein is shown in *B. jararaca*, since the protein sequence described in UNIPROT databank (http://www.uniprot.org) was derived from DNA data. Moreover, *α*-PLI was also possible to be identified, and about 48% of its sequence was obtained (data not shown). As seen for Bj46a, all of the 122 amino acids identified showed identity to the corresponding sequence present in the databank. It is noteworthy the high variability of PLIs found in juvenile or adult plasma, probably not only due to different glycosylation pattern but also to the amino acid sequence, as illustrated by spots 14 and 20 (Figure 2), which are similar to *Trimeresurus flavoviridis *PLIs. This peculiarity is related to the high incidence of PLIs isoforms present in snake plasma, showing the physiological importance of these inhibitors for the physiology of these animals [60, 61].

The role played by PLIs has been the physiological protection of snakes against accidental envenomation or due to the feeding habits of *ophiophagous* specimens [8, 28, 60]. In the last two decades, the number of reports on endogenous PLIs in the plasma of snakes has increased, motivated by the need to develop potentially selective inhibitors for human PLA_2_.

Snake venom PLA_2_ exhibits a wide variety of pharmacological effects and is involved in the envenomation pathophysiology, presenting myotoxic and neurotoxic activities [62]. Antunes et al. [57] demonstrated that newborn *B. jararaca *venom shows catalytic PLA_2_ activity almost twice higher than that of the adult venom, and our results showed that the same occurs regarding *γ*-PLI, indicating a connection between venom and plasma components. In addition, besides the antivenom role of PLI, these proteins can be a favorable therapeutic approach in the treatment of inflammatory processes, once *γ*-PLI has been studied as a potential model for the development of selective inhibitors of proinflammatory PLA_2_ in humans [60, 63].

In short, the results showed that there are some differences in plasma protein composition between juvenile and adult *B. jararaca *and that these differences could be related to the ontogenetic variation of the venom composition. This is the first comparative study of protein profiles of juvenile and adult snake plasma. This approach is important for a better understanding of the ontogenetic development of *B. jararaca*. Moreover, associated with the knowledge of ontogenetic changes in venom composition and snakebite clinical reports, the differences identified could be used for the development of more specific antivenoms. It has been suggested that antiophidian serum could be enriched with natural antitoxins in order to increase the serum ability to neutralize snake venom [63]. Thus, the results presented here in this paper can contribute to the knowledge of antivenom mechanisms against ophidian accidents.

## Figures and Tables

**Table 1:** Number of matches and spots present at different levels in juvenile and adult *B. jararaca* plasma. 2D electrophoresis were analyzed by ImageMaster Platinum 7.0 software (GE Healthcare).

	Number of matches	Spots showing quantitative	
		variation	
		Exclusive spots	Increased spots
Juvenile *B. jararaca *	1,250	18	5
Adult *B. jararaca *		16	6

		Total: 45	

**Table:** (a)

Juvenile *Bothrops jararaca* snake					
Spot number^a^	Protein name (organism)	Score	Protein accession number^b^	Peptide sequences^c^	Volume^d^ (%)
1	*γ*-phospholipase A_2_ inhibitor (*Bothrops jararaca*)	556	gi∣157885066	KCIDIVGHR KNCFSSSICKL SCDFCHNIGK VFLEISSASLSVR HEHFPGDIAYNLK LGQIDVNIGHHSYIR DCDGYQQECSSPEDVCGK CIDIVGHRHEHFPGDIAYNLK	1.6137

14	*γ*-phospholipase A_2_ inhibitor subunit B (*Trimeresurus flavoviridis*)	120	gi∣155676753	RACCVGDECK GCATESLCTLLQK	1.6347

16	*γ*-phospholipase A_2_ inhibitor (*Bothrops jararaca*)	467	gi∣157885066	KCIDIVGHR NCFSSSICK VFLEISSASLSVR HEHFPGDIAYNLK TVHKNCFSSSICK LGQIDVNIGHHSYIR DCDGYQQECSSPEDVCGK KCIDIVGHRHEHFPGDIAYNLK	0.5292

20	*γ*-phospholipase A_2_ inhibitor subunit B (*Trimeresurus flavoviridis*)	284	gi∣155676753	ACCVGDECK RACCVGDECK DTENQCLSLTGK GCATESLCTLLQK	0.5687

22	*γ*-phospholipase A_2_ inhibitor (*Bothrops jararaca*)	496	gi∣157885068	INCCEK KCIDIVGHR GRINCCEK NCFSSSICK VFLEISSASLSVR HEHFPGDIAYNLK TVHKNCFSSSICK LGQIDVNIGHHSYIR KCIDIVGHRHEHFPGDIAYNLK	3.5287

39	C3 complement (*Naja naja*)	221	gi∣399269	RVGLVAVDK IWDTIEK IQKPGAAMK GIYTPGSPVR IKLEGDPGAR AVYVLNDKYK EYVLPSFEVR	0.1644

44	Albumin (*Trimeresurus flavoviridis*)	92	gi∣56790036	LVEDIQNDHIIQ IIPQAPTSNLIEITKR	0.3461

**Table:** (b)

Adult *Bothrops jararaca* snake					
Spot number^a^	Protein name (organism)	Score	Protein accession number^b^	Peptide sequences	Volume^c^ (%)
7	*γ*-phospholipase A_2_ inhibitor (*Bothrops jararaca*)	511	gi∣157885066	KCIDIVGHR NCFSSSICK VFLEISSASLSVR HEHFPGDIAYNLK LGQIDVNIGHHSYIR DCDGYQQECSSPEDVCGK KCIDIVGHRHEHFPGDIAYNLK	0.5813

16	*γ*-phospholipase A_2_ inhibitor (*Bothrops jararaca*)	470	gi∣157885066	KCIDIVGHR NCFSSSICK SCDFCHNIGK VFLEISSASLSVR HEHFPGDIAYNLK LGQIDVNIGHHSYIR DCDGYQQECSSPEDVCGK	0.0990

18	Transferrin (*Lamprophis fuliginosus*)	220	gi∣108792441	IVWCAVGK VCTFHTHDW EADAITLDGGHIYTAGK	0.4829

24	Transferrin (*Lamprophis fuliginosus*)	232	gi∣108792441	LVLEQQK IVWCAVGK VCTFHTHDW EADAITLDGGHIYTAGK	0.2124

29	C3 complement (*Naja naja*)	363	gi∣399269	VGLVAVDK LEGDPGAR IWDTIEK IQKPGAAMK GIYTPGSPVR IKLEGDPGAR DTCMGTLVVK AVYVLNDKYK EYVLPSFEVR	0.2256

38	*α*-phospholipase A_2_ inhibitor precursor (*Bothrops jararaca*)	383	gi∣167547111	LYVTNK REFANLR KNFEALR RSFGSGSER GAFLTVHKA KAFANVLER KVLNSLIDALMHLQRE QICEQAEGHIPSPQLENHNK	0.1067

40	*β*-Actin (*Rachycentron canadum*)	867	gi∣161376754	AGFAGDDAPR DLTDYLMK GYSFTTTAER EITALAPSTMK AVFPSIVGRPR DSYVGDEAQSKR IWHHTFYNELR QEYDESGPSIVHR LDLAGRDLTDYLMK SYELPDGQVITIGNER EEEIAALVVDNGSGMCK VAPEEHPVLLTEAPLNPK DLYANTVLSGGTTMYPGIADR TTGIVMDSGDGVTHTVPIYEGYALPHAILR	0.0060

41	Antihemorrhagic factor Bj46a (*Bothrops jararaca*)	68	gi∣48428681	YALNVIKN EGHAHSHLIQQHVEK NCPKCPILLPSNNPQVVDSVEYVLNKHNEK HNEKLSDHVYEVLEISR GDLECDEKDAKEWTDTGVR IMFNVDTFKEDVFAK LSDHVYEVLEISR VPVAFVK ELPKDISDR VHHFEL EWTDTGVR	0.09264

43	Albumin (*Trimeresurus flavoviridis*)	234	gi∣56790036	ECFDTK YGINDCCAK LVEDIQNDHIIQ QLCHCCDSSFISR LEDHVQCLHTGEEQLK	0.0497

44	Albumin (*Trimeresurus flavoviridis*)	235	gi∣56790036	FIETHEK NNCDNYK LVEDIQNDHIIQ QLCHCCDSSFISR LEDHVQCLHTGEEQLK	0.3964

^a^Spot number refers to that shown in Figure 2.

^b^NCBI accession number.

^c^Obtained by MS/MS analysis.

^d^ *P* < 0.05.
